# Safety and Efficacy of CD19-Targeted Chimeric Antigen Receptor (CAR) T-cells Generated Using DNA Transposon Systems: A Meta-Analysis

**DOI:** 10.7759/cureus.81930

**Published:** 2025-04-08

**Authors:** Anugya Rajput, Syed Ammar Sajjad, Anup Kumar, Kulwant Singh

**Affiliations:** 1 Stem Cell Research Center, Department of Hematology, Sanjay Gandhi Postgraduate Institute of Medical Sciences, Lucknow, IND; 2 Department of Biostatistics and Health Informatics, Sanjay Gandhi Postgraduate Institute of Medical Sciences, Lucknow, IND

**Keywords:** car t-cell, dna transposon, hematological malignancies, piggybac, sleeping beauty

## Abstract

Chimeric antigen receptor (CAR) T-cell therapy has emerged as a potentially curative approach for hematological malignancies. The DNA transposon system represents a non-viral approach to CAR T-cell generation, offering several advantages including low immunogenicity, scalability, and cost-effectiveness over existing methods. Despite significant clinical advances, no meta-analysis has been conducted to evaluate the safety and efficacy of DNA transposon-generated CAR T-cells. This meta-analysis aims to evaluate the efficacy and safety of DNA transposon-generated CAR T-cell therapy across B-cell malignancies. A systematic literature search was conducted through databases, PubMed, Google Scholar, OpenAlex, and Semantic Scholar, from 2012 to January 2024. A total of seven studies encompassing 110 patients were found eligible. The pooled analysis demonstrated an overall response rate of 75%, with a complete response achieved in 66% of patients. Moreover, 49% of patients demonstrated progression-free survival (PFS) with a median follow-up of 30 months, and 53% of patients achieved negative measurable residual disease (NMRD) remission. Notably, few patients experienced cytokine release syndrome (CRS) of grades 1-2; however, neurotoxicity was not described as a prevalent side effect. DNA transposon-generated CD19 CAR T-cell therapy demonstrates promising efficacy in B-cell malignancies, with favorable safety profiles. However, the outcomes of this meta-analysis underscore the need for further clinical development.

## Introduction and background

The advent of chimeric antigen receptor (CAR) T-cell therapy has revolutionized the treatment landscape for hematological malignancies, offering promising outcomes for patients with relapsed/refractory diseases. CARs are synthetic immunoreceptors that enable T-cells to target and eliminate tumor cells in a major histocompatibility complex (MHC)-independent manner [1]. CAR consists of an antigen-binding domain linked to hinge, transmembrane, co-stimulatory, and signaling domains. The second-generation CAR includes a single co-stimulatory domain, while two co-stimulatory domains are incorporated in third-generation CARs. Currently, CD19-directed CAR T-cell therapy is being explored particularly in the clinical management of B-cell malignancies including acute lymphoblastic leukemia (ALL) and non-Hodgkin lymphoma (NHL) [2]. However, challenges such as cytokine release syndrome (CRS), neurotoxicity, and complex manufacturing processes have underscored the need for continuous refinement and optimization of CAR T-cell therapies [3-5]. The FDA-approved CAR T-cell therapies rely on lentiviral or gamma retroviral vectors for CAR gene transfer and stable expression. However, viral gene delivery systems are associated with profound safety concerns as they tend to integrate the CAR gene into the promoter or coding regions of highly expressed genes or tumor-associated genes. This integration leads to insertional mutagenesis potentially causing secondary malignancies [6-8]. Elevated immunogenicity and limited size insert are additional disadvantages associated with the viral system. In particular, the production cost of viral vectors and regulatory requirements related to their clinical use further restrict the goal of making CAR T-cell therapy available to patients in middle- or low-income countries.

The DNA transposon system offers a promising alternative to viral-based methods for CAR T-cell generation [9]. DNA transposons are mobile genetic elements that move within the genome via a DNA intermediate using a “cut-and-paste” mechanism. In a non-viral gene delivery system, the target gene integrates into chromosomes through a precise recombinase-mediated process, enabling the stable and long-term expression of the gene of interest [10]. This approach enables genomic integration without the need for viral vectors with the advantage of stable transgene expression, low immunogenicity, enhanced safety profile, potentially reduced manufacturing costs, and scalability [11]. The DNA transposon system employs a two-component vector system: one vector carries the CAR gene flanked by inverted terminal repeats (ITRs), while the other encodes a transposase enzyme that binds to the ITRs and facilitates genomic integration. To date, two DNA transposon-based gene delivery systems have been utilized to deliver the CD19-CAR gene, the Sleeping Beauty (SB) and the PiggyBac (PB) transposon systems. The safety and efficacy of anti-CD19 CAR T-cells manufactured using the SB transposon system (SB-CD19-CAR T-cells) and PB (PB-CD19-CAR T-cells) have been evaluated in Phase I/II clinical trials using second-/third-generation CAR constructs [12-18]. However, meta-analyzed pooled data on the clinical outcomes of these CAR T-cell therapies is still lacking. Therefore, we undertook this meta-analysis to comprehensively evaluate the safety and efficacy profile of DNA transposon-CD19 CAR T-cells in the treatment of B-cell malignancies.

The study was registered with PROSPERO (CRD42024507597). This article was originally posted as a preprint on Research Square in June 2024 under the title “A Meta-Analysis on the Safety and Efficacy of CD19-Directed CAR T-cells Generated Using Sleeping Beauty Transposon System.”

## Review

Materials and methods

The meta-analysis was designed following the Preferred Reporting Items for Systemic Reviews and Meta-Analyses (PRISMA) 2020 guidelines.

Study selection

The articles were manually retrieved from PubMed, Google Scholar, Semantic Scholar, and OpenAlex by searching all the entries from January 2012 till January 2024. The PRISMA guidelines were followed throughout the search for articles. Different journal titles, abstracts, and full-text articles were found. Boolean operators AND/OR were used for the search strategy. Multiple filters were also implied to make the search specific for articles. Two researchers (AR and SAS) carried out the same search independently, and in case of disagreement, a third researcher (KS) was involved to discuss the result and reach an agreement. We searched peer-reviewed journals and publications for relevant literature in order to meet the inclusion criteria. We tried to "include" or "exclude" pertinent studies based on the inclusion and exclusion criteria. Among all studies, seven articles were taken into account for the final review and analysis. Research that failed the eligibility requirements for screening was classified as "dispute" or "exclusion." Prior to removing a study from the literature, the exclusion criteria were presented.

Inclusion Criteria

Studies focused on the diagnostic value of CD19-directed CAR T-cells generated using the DNA transposon systems; the studies included adult patients and pediatric patients who have hematological malignancies like ALL, NHL, and chronic lymphocytic leukemia (CLL). The clinical diagnostic criteria for safety were CRS and immune effector cell-associated neurotoxicity syndrome (ICANS), whereas complete response (CR), overall response rate (ORR), progression-free survival (PFS), and negative measurable residual disease (NMRD) were included as a criterion of efficacy.

Exclusion Criteria

Papers published in languages other than English were not considered for the article. Animal studies, systematic reviews and meta-analyses, narrative reviews, and case reports were excluded.

Quality assessment

Two investigators (AR and SAS) independently evaluated the methodological quality of each study by applying the diagnostic accuracy tool Quality Assessment of Diagnostic Accuracy Studies version 2 (QUADAS-2) from the Review Manager software (The Cochrane Collaboration, London, UK) to assess the quality of all seven included articles. The QUADAS-2 scale includes four parts: case selection, trial to be evaluated, the gold standard, and case process and progress.

Data extraction

PRISMA guidelines were followed to perform the meta-analysis. A thorough search of various electronic databases, including PubMed, Google Scholar, Semantic Scholar, and OpenAlex, was conducted as part of the methodology. The studies highlighting the safety and efficacy profile as the therapeutic outcome of DNA transposon-generated CAR T-cell therapy were included. The search strategy included the following keywords: CD19, hematological malignancies, Sleeping Beauty, PiggyBac, DNA transposon, chimeric antigen receptor, clinical trial, and non-viral gene transfer. These keywords were used to identify relevant research articles focused on CD19-directed CAR T-cell therapies generated using DNA transposon systems and their applications in hematologic malignancies. In addition, the studies were indexed using a set of MeSH headings, including chimeric antigen receptor T-cell therapy, CD19 antigen, DNA transposons, gene transfer techniques, immunotherapy, meta-analysis, and T-lymphocytes, as well as safety and efficacy. There were two phases to the article screening process. The titles and abstracts of every article found in the chosen electronic databases were examined during the first screening phase. Articles that passed the first round of screening underwent screening for the second round. For every eligible article, uniform data extraction tables with information on the first author, year of publication, study design, country of study, location, sample size, and related factors were tabulated. The research data was independently extracted by two researchers. If the extraction results of the two were inconsistent, the third researcher and the first two jointly studied and decided.

Statistical analysis

This study was a proportional meta-analysis. The heterogeneity of the included articles was determined to select the appropriate statistical model to help reduce errors during data merging. The heterogeneity between the included studies was evaluated by calculating the chi-squared test value and the I^2^ statistics. If I^2^ ≤ 50%, p ≥ 0.05, the heterogeneity of the included studies was deemed small, and the fixed- or common-effect model was used to merge the statistical data. If I^2^ > 50%, p < 0.05, the heterogeneity was significant, and data were merged by the random-effects model. The indexes included were CRS, ICANS, CR, ORR, PFS, and NMRD. Heterogeneity is addressed using Cochran’s Q statistic and I^2^ measure. This describes the percentage of variation in effect estimate that is due to heterogeneity rather than sampling error (chance). The risk of biases was calculated through the Deeks funnel plot and Egger test of each parameter by AR and SAS independently.

The “metaprop” function from the “meta” package calculates the total effect size when there are several events (safety and efficacy) and several samples (n) in the proportion-type data. Among the methods for calculating the effect size in proportion-type data, the method based on logit transformation and then back transform was used (the logit transformation and Clopper-Pearson method). When the assumptions of the statistical model are properly applied for consistency and considering the symmetry and distribution of the data, it is preferable to transform (log transformation or logit transformation) the proportion-type data because this produces conservative results through the transformation. The inverse variance method is a basic method of meta-analysis, which utilizes the inverse variance of the applicable study when calculating the weights of individual studies, and the tau value, which is the tween-study variance, was calculated using the DerSimonian-Laird estimator in a random-effects model. The software codes used to conduct the meta-analysis are provided in Supplemental material 1.

Results

Selection of Eligible Articles

**Figure 1:**
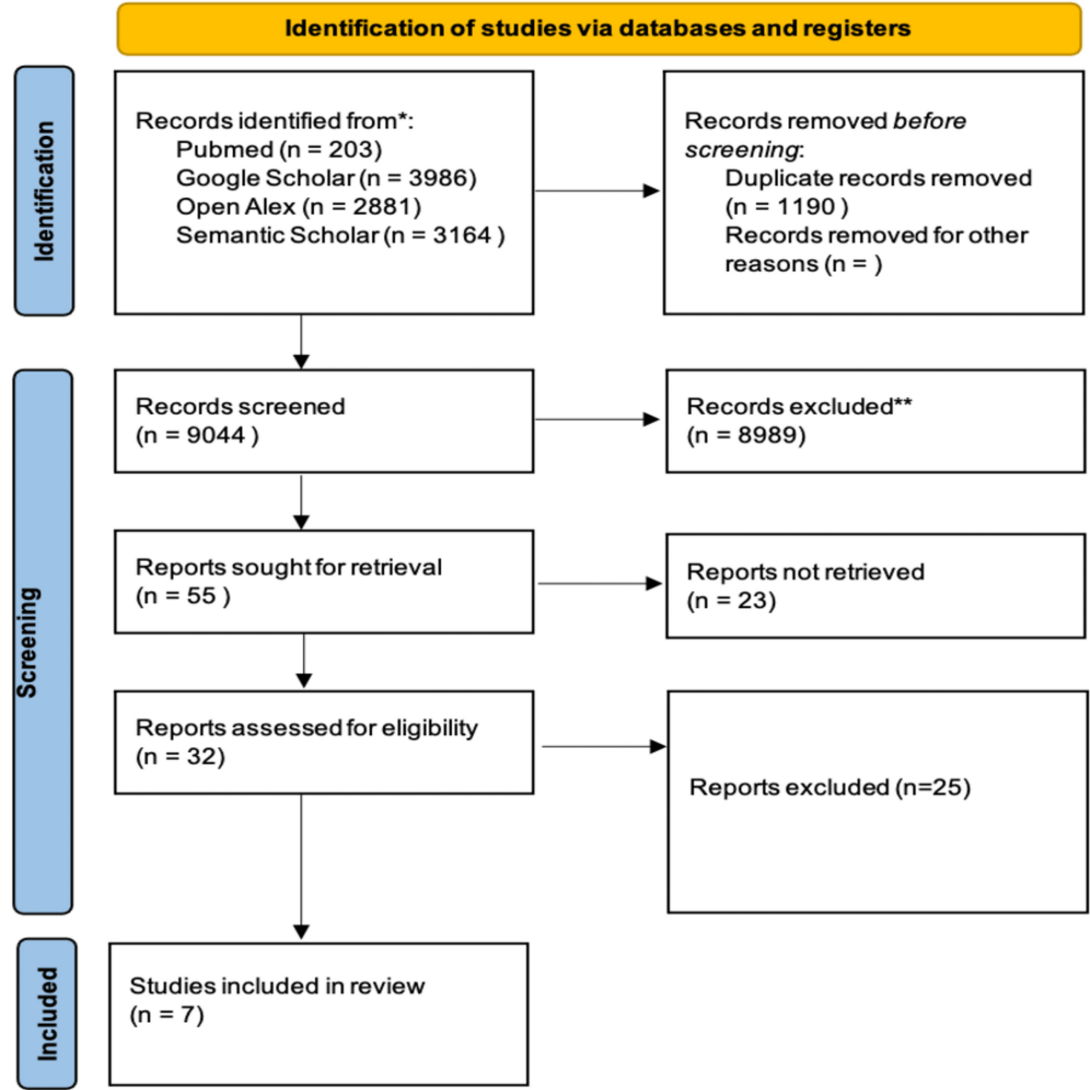
PRISMA flowchart showing the study selection process PRISMA: Preferred Reporting Items for Systematic Reviews and Meta-Analyses

A comprehensive search of selected bibliographic databases retrieved 9,044 citations after the removal of duplicate records. Screening of the titles and abstracts identified 32 potential studies, which were considered eligible. Finally, seven articles were included in the meta-analysis based on the inclusion and exclusion criteria. Figure 1 presents the study flow diagram as per the criteria set up by PRISMA 2020.

Study Characteristics

A total of 110 patients were included in the present analysis of which 76.3% (84/110) were B-cell acute lymphoblastic leukemia (B-ALL), 2.72% (3/110) were CLL, and 23.2% (23/110) were B-cell non-Hodgkin lymphoma (B-NHL) patients. The outcomes of all the studies were published between 2012 and 2024. The study characters and interventions in the included studies are provided in Tables 1, 2. Quality assessment of the clinical trial was carried out using the QUADAS-2 assessment tool that revealed most studies met the quality of the inclusion criteria. The details of the risk of bias assessment of each variable are presented in Supplemental material 2.

**Table 1:** Baseline characteristics CAR: chimeric antigen receptor; B-ALL: B-cell acute lymphoblastic leukemia; CLL: chronic lymphocytic leukemia; DLBCL: diffuse large B-cell lymphoma; HSCT: hematopoietic stem cell transplantation; NHL: non-Hodgkin lymphoma; MCL: mantle cell lymphoma; FL: follicular lymphoma; TNC: total nucleated cell

Author	Clinical trial	Clinical trial phases	Study center	CAR construct signaling/generation	Origin of CAR T-cells	Patient population	Prior therapy (Allo/Auto HSCT)	Sample size	Conditioning chemotherapy	Dosage of Sleeping Beauty CAR T-cell
Singh et al., 2022 [12]	NCT02807883	Phase 1	M.D. Anderson Cancer Center, USA	CD19RCD8CD28z	Autologous	B-ALL = 8, NHL = 4, CLL = 2	Allogeneic = 10	14 adults	Fludarabine 30 mg/m^2^ and cyclophosphamide 500 mg/m^2^	DL -1: ≤1 x 10^5^/kg; DL +1: >1 x 10^5^/kg but ≤1 x 10^6^/kg; DL +2: >1 x 10^6^/kg but ≤1 x 10^7^/kg; DL +3: >1 x 10^7^/kg but ≤1 x 10^8^/kg; DL +4: >1 x 10^8^/kg but ≤ 1 x 10^9^/kg
Rambaldi et al., 2022 [13]	NCT03389035	Phase I/IIb	Tettamanti Research Center, Italy	CD19RCD8CD28OX40z	Allogeneic	B-ALL = 20	NA	20 adults	NA	7.5 x 10^6^/kg and 15 x 10^6^/kg transduced CAR + T-cells/kg
Srour et al., 2020 [14]	NCT01492036	Phase 1	M.D. Anderson Cancer Center, USA	CD19RCD8CD28z	Autologous	DLBCL = 4, DLBCL CNS lymphoma = 1, FL = 1, MCL = 1	Autologous = 7	7 adults	Carmustine, etoposide, cytarabine, and melphalan	NA
Bishop et al., 2021 [15]	ACTRN12617001579381	Phase 1	Westmead Institute for Medical Research, Australia	CD19IgG1CD284-1BBz	Allogeneic	B-ALL = 8, DLBCL = 1, Burkitt = 1	Allogeneic = 10	10 adults	Fludarabine (25 mg/m^2^/day) and cyclophosphamide (250 mg/m^2^/day)	1 x 10^7^ TNC/m^2^ (Flu/Cy), 5 x 10^7^ TNC/m^2^ (Cy), 5 x 10^7^ TNC/m^2^ (Flu/Cy), 1 x 10^8^ TNC/m^2^ (Cy)
Kebriaei et al., 2016 [16]	Autologous, NCT00968760; allogeneic, NCT01497184; long-term follow-up, NCT01492036	Phase 1	M.D. Anderson Cancer Center, USA	CD19RCD8CD28z	Autologous	NHL = 9, B-ALL = 17	Autologous = 7, allogeneic = 19	26 adults	NA	NA
Lussana et al., 2022 [17]	NCT03389035	Phase I/II	Tettamanti Research Center, Italy	CD19RCD8CD28OX40z	Allogeneic	B-ALL = 27	Allogeneic = 27	4 pediatric, 23 adult	Fludarabine (30 mg/m^2^/day x 4 days) and cyclophosphamide (500 mg/m^2^/day x 2 days)	1 × 10^6^, 3 × 10^6^, 7.5 × 10^6^, and 15 × 10^6^ transduced CAR + T-cells/kg
Kebriaei et al., 2017 [18]	NCT02529813	Phase 1	M.D. Anderson Cancer Center, USA	CD19RCD8CD28z	Autologous	CLL = 1, B-ALL = 4, DLBCL = 1	Allogeneic = 4	6 adults	NA	Starting at ≤10^5^ (dose level, DL -1), >10^5^ but ≤10^6^ (dose level, DL 1), and then escalating in cohorts of 3 to the dose of >10^8^ but ≤10^9^ CD3 + CAR + cells/kg (DL 4) but ≤10^9^ CD3 + CAR + cells/kg (DL 4)

**Table 2:** Clinical outcomes of DNA transposon-generated anti-CD19 CAR T-cell therapy B-ALL: B-cell acute lymphoblastic leukemia; CLL: chronic lymphocytic leukemia; DLBCL: diffuse large B-cell lymphoma; CRS: cytokine release syndrome; ICANS: immune effector cell-associated neurotoxicity syndrome; HSCT: hematopoietic stem cell transplantation; CR: complete remission; MCL: mantle cell lymphoma; FL: follicular lymphoma; NHL: non-Hodgkin lymphoma; ORR: overall response rate; CAR: chimeric antigen receptor

Author and year	Median age (years)	Patient population	Prior line of therapy	Median follow-up	Efficacy						Safety	
					Overall response	Complete response	Progression-free survival	Negative measurable residual disease (NMRD)	Positive measurable residual disease (PMRD)	CAR T persistence	Number of patients negative for grade 1-2 CRS	Number of patients negative for grade 1-2 ICANS
Singh et al., 2022 [12]	40	B-ALL = 8, DLBCL = 4, CLL = 2	4	14.8 months	57%, B-ALL: 50%, DLBCL: 50%	B-ALL: 38% (3/8) at 1 month, DLBCL: 50% (2/4) CR at 1 month	21% in 1 year, B-ALL: 13%, DLBCL: 50%	B-ALL: 13% (1/8)	16.6% (2/12), B-ALL = 1/8, DLBCL = 1/4	4.5 years	11	14
Rambaldi et al., 2022 [13]	NA	B-ALL = 20	NA	NA	71.4% (6 months)	76.2% in 28 days	NA	81.30%	NA	12 months	15	15
Srour et al., 2020 [14]	52	DLBCL = 4, DLBCL CNS lymphoma = 1, FL = 1, MCL = 1	Median = 3	4.7 years	86% (5 years)	(4/7)	71% (5 years)	NA	(3/7)	4.5 years	7	7
Bishop et al., 2021 [15]	36	B-ALL = 8, DLBCL = 1, Burkitt = 1	Median = 5	18.0 months	100% (9/9)	100% (9/9)	NA	(6/9)	NA	4.9 months	5	9
Kebriaei et al., 2016 [16]	NA	B-ALL = 17, NHL = 9	NA	NA	Autologous HSCT ORR (3 years): 100%, allogenic HSCT ORR (2 years): 49%	Autologous HSCT (3.4 years) (6/7), allogenic HSCT (1.5 years) (6/19)	3 years autologous: 86%, 2 years allogeneic: 45%	NA	NA	NA	NA	NA
Lussana et al., 2022 [17]	NA	B-ALL = 27	Median: 4 (range: 2–8)	2.8 years	71.4% at 6 months	18/27 (66.7%)at single CAR T dose; 16/21 (76.2%) at two highest doses on day 28	NA	14/27 (77.8%) at single dose; 13/21 (81.3%) at two highest doses	NA	NA	9	2/21 at two highest doses (grade 3)
Kebriaei et al., 2017 [18]	41	CLL = 1, B-ALL = 4, DLBCL = 1	NA	3 months	NA	NA	NA	NA	NA	NA	4	NA

Assessment of Heterogeneity

A chi-squared and DerSimonian-Laird random-effects meta-analysis was conducted to make direct comparisons. Cochran's Q test was used to assess statistically significant heterogeneity not explained by chance, whereas I^2^ was used to quantify the total observed variability due to between-study heterogeneity. I^2^ values range from 0% (no heterogeneity) to 100% (highest heterogeneity). I^2^ values > 50% were considered to indicate substantial heterogeneity. Statistical significance was established with an α level of 0.05 for primary analyses. Details for heterogeneity assessment are described in Supplemental material 3. Heterogeneity analysis revealed that baseline demographics for all parameters such as median age, therapy before CAR infusion (allogeneic and autologous hematopoietic stem cell transplantation (HSCT)), doses of CAR T-cells, prior line of therapy, median follow-up duration, and efficacy that includes CR, ORR, PFS, and NMRD along with safety, CRS, and ICANS were comparable across all the included studies.

Efficacy of DNA Transposon-CD19 CAR T-cells

**Figure 2:**
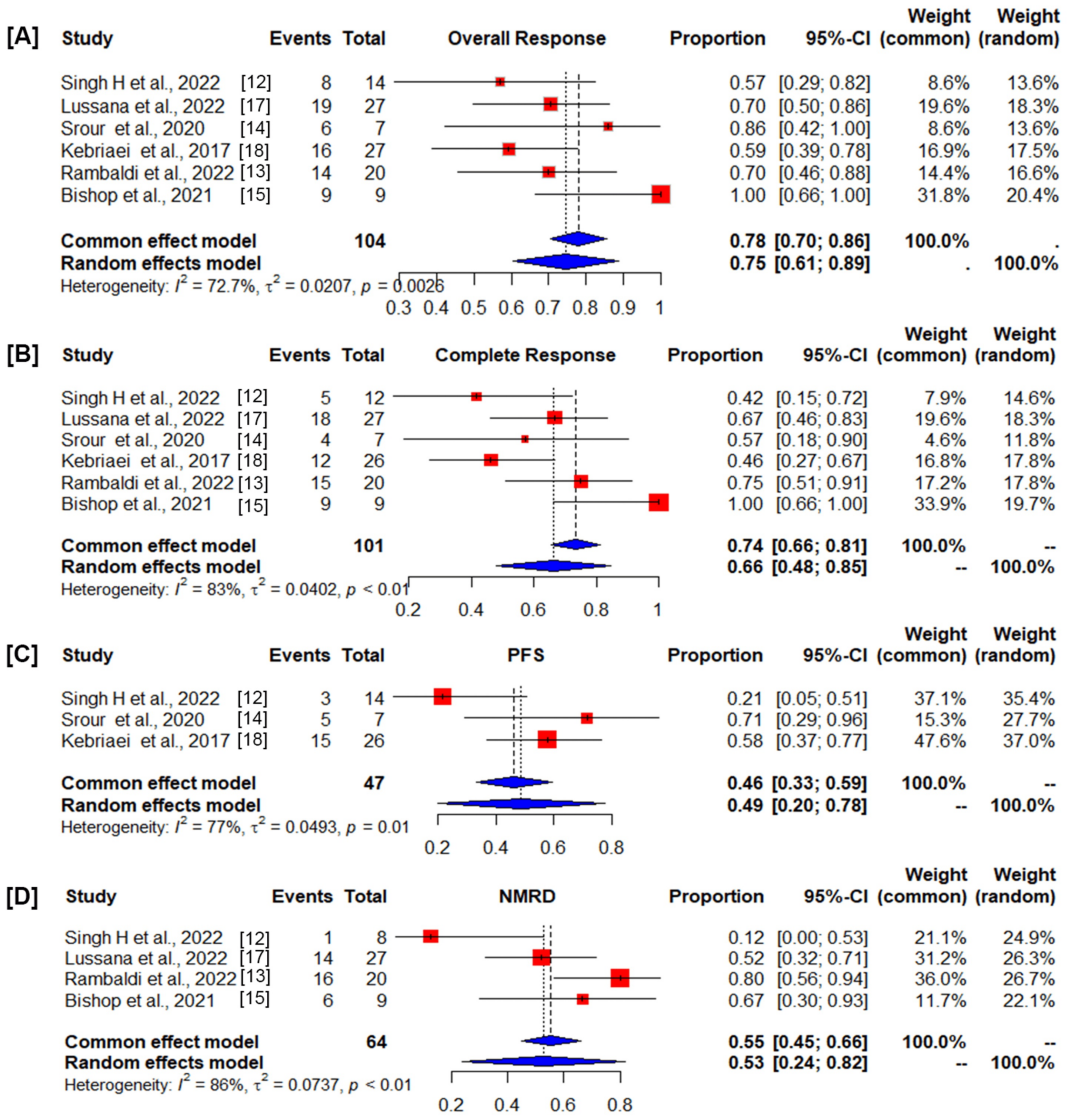
Forest plot showing (A) overall response rate (ORR), (B) complete response (CR), (C) progression-free survival (PFS), and (D) negative measurable residual disease (NMRD) in patients with B-cell malignancy. Squares represent the event rates (square size reflects the study‐specific statistical weight), horizontal lines represent the 95% CI, and diamonds represent the pooled estimate based on a random‐effects model

Having evaluated the methodological consistency of the included studies, we next assessed the efficacy profile of the DNA transposon-CD19 CAR T cell therapy. A total of 110 patients were included in the present study of which 76.3% (84/110) were B-ALL, 2.72% (3/110) were CLL, and 23.2% (23/110) were B-NHL patients, and a majority of the patients in the cohorts either relapsed after autologous/allogeneic bone marrow transplantation or received CAR T-cell therapy after HSCT (Table 1). In these studies, infused CAR T-cell products either expressed second-generation (CD19RCD8CD28z) or third-generation (CD19RCD28OX40z) (Table 1). The single-chain variable fragment (ScFV) was derived from FMC63. The co-stimulatory domain was CD28 (second-generation CAR) or CD28 and OX40 (third-generation CAR). In all the studies, CAR gene delivery was carried out using the SB transposon system except for one study that used the PB transposon system [15]. CAR T-cell dosages varied considerably across the different studies ranging from 1 x 10^5^ to 5 x 10^9^ cells/kg body weight (Table 1). Cyclophosphamide in combination with fludarabine was the most frequently used lymphodepleting chemotherapy regimen; however, other chemotherapeutic drugs were also administered (Table 1). The pooled estimates of overall response were 0.78 (95% CI, 0.70-0.86) in the common-effect model and 0.75 (95% CI, 0.61-0.89) in the random-effects model. The value I^2^ = 72.7% (95% CI, 60%-89.2%), p = 0.0028, which shows the heterogeneity among 104 evaluable patients, was significant (Figure 2). The pooled estimates of CR were 0.74 (95% CI, 0.66-0.81) in the common-effect model and 0.66 (95% CI, 0.48-0.85) in the random-effects model (Figure 2). The value I^2^ = 83% (95% CI, 63.6%-91.8%), p < 0.0001, which shows heterogeneity among 101 evaluable patients, was significant (Figure 2). PFS was 0.46 (95% CI, 0.33-0.59) in the common-effect model and 0.49 (95% CI, 0.20-0.78) in the random-effects model (Figure 2). The value I^2^ = 77% (95% CI, 25%-92.9%), p < 0.013, which shows the heterogeneity among 47 evaluable patients, was significant. The cumulative incidence of NMRD remission was 0.55 (95% CI, 0.45-0.66) in the common-effect model and 0.53 (95% CI, 0.24-0.82) in the random-effects model (Figure 2). The value I^2^ = 86.2% (95% CI, 66.2%-94.3%), p < 0.0001, which shows the heterogeneity among patients, is significant (Figure 2). For the pooled estimated overall response, CR, PFS, and NMRD remission, the heterogeneity is greater than 50% and the p-value is less than 0.05; hence, the random-effects model is applied to merge the statistical data.

Safety Assessment DNA Transposon-CD19 CAR T-cells

**Figure 3:**
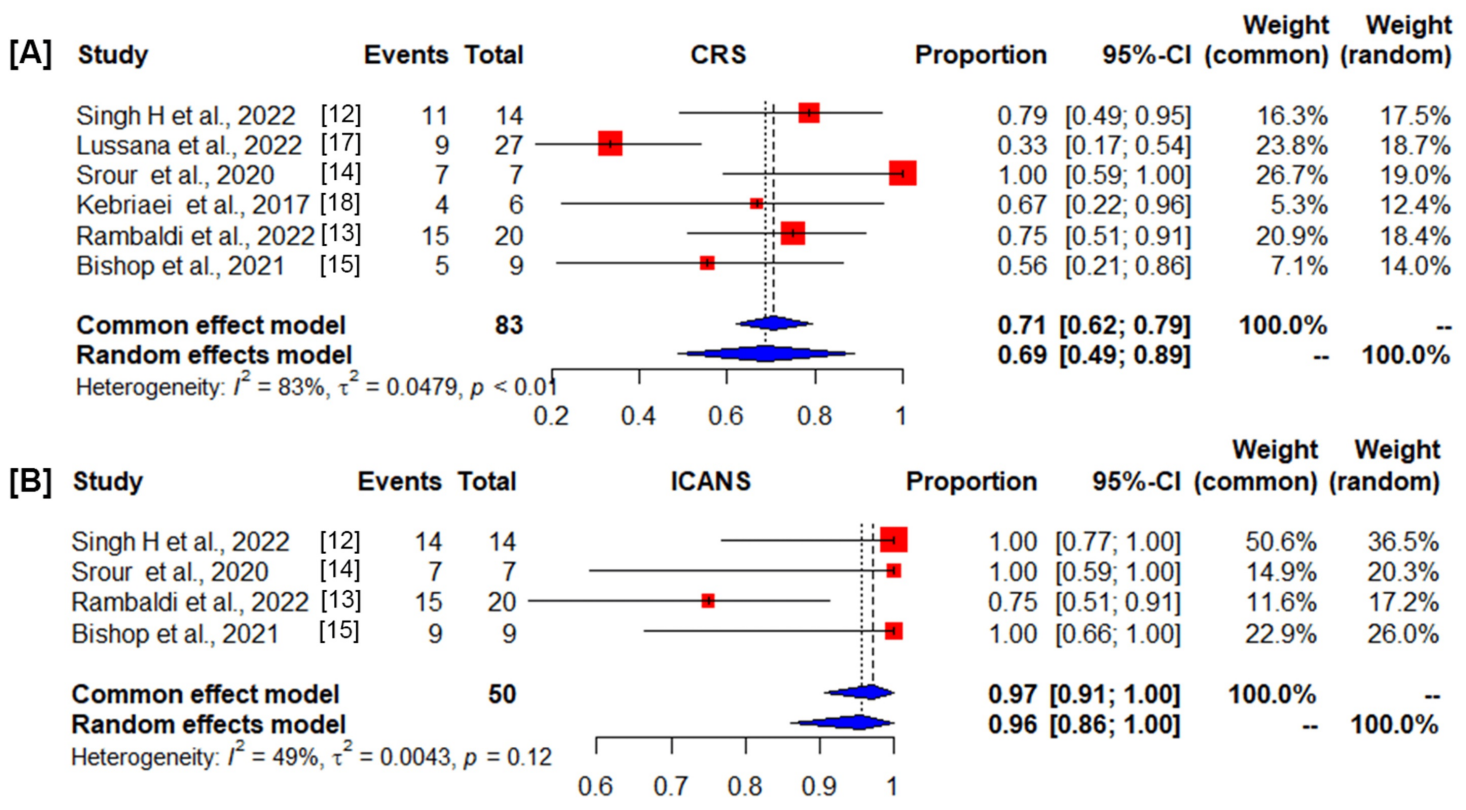
Forest plot showing the number of patients negative for adverse events of DNA transposon (DT)-CD19 chimeric antigen receptor (CAR) T-cell therapy, (A) cytokine release syndrome (CRS), and (B) immune effector cell-associated neurotoxicity syndrome (ICANS). Squares represent the event rates (square size reflects the study‐specific statistical weight), horizontal lines represent the 95% CI, and diamonds represent the pooled estimate based on a random‐effects model

Among 110 patients evaluable for safety, the pooled cumulative incidence rate of the number of patients negative for grade 1/2 CRS estimates was 0.71 (95% CI, 0.62-0.79) in the common-effect model and 0.69 (95% CI, 0.49-0.89) in the random-effects model. The value I^2^ = 83% (95% CI, 65.4%-92.1%), p<0.0001, shows the heterogeneity was significant (Figure 3). CRS of grade 3 was not reported among patients treated with DNA transposon-CAR T-cells. The cumulative incidence rate of the number of patients negative for grade 1/2 ICANS estimates was 0.97 (95% CI, 0.91-1.00) in the common-effect model and 0.96 (95% CI, 0.86-1.00) in the random-effects model. The heterogeneity I^2^ = 49.1% (95% CI, 0.0%-83.1%), p = 0.12 (Figure 3), suggested that DNA transposon-CD19 CAR T-cell therapy retains a high safety profile. For the pooled estimated CRS, the heterogeneity is greater than 50% and the p-value is less than 0.05; hence, the random-effects model is applied. For pooled ICANS, I^2^ ≤ 50% and p ≥ 0.01, the heterogeneity of the included studies was deemed small, and the common-effect model was used to merge the statistical data.

Assessment of Reporting Biases

**Figure 4:**
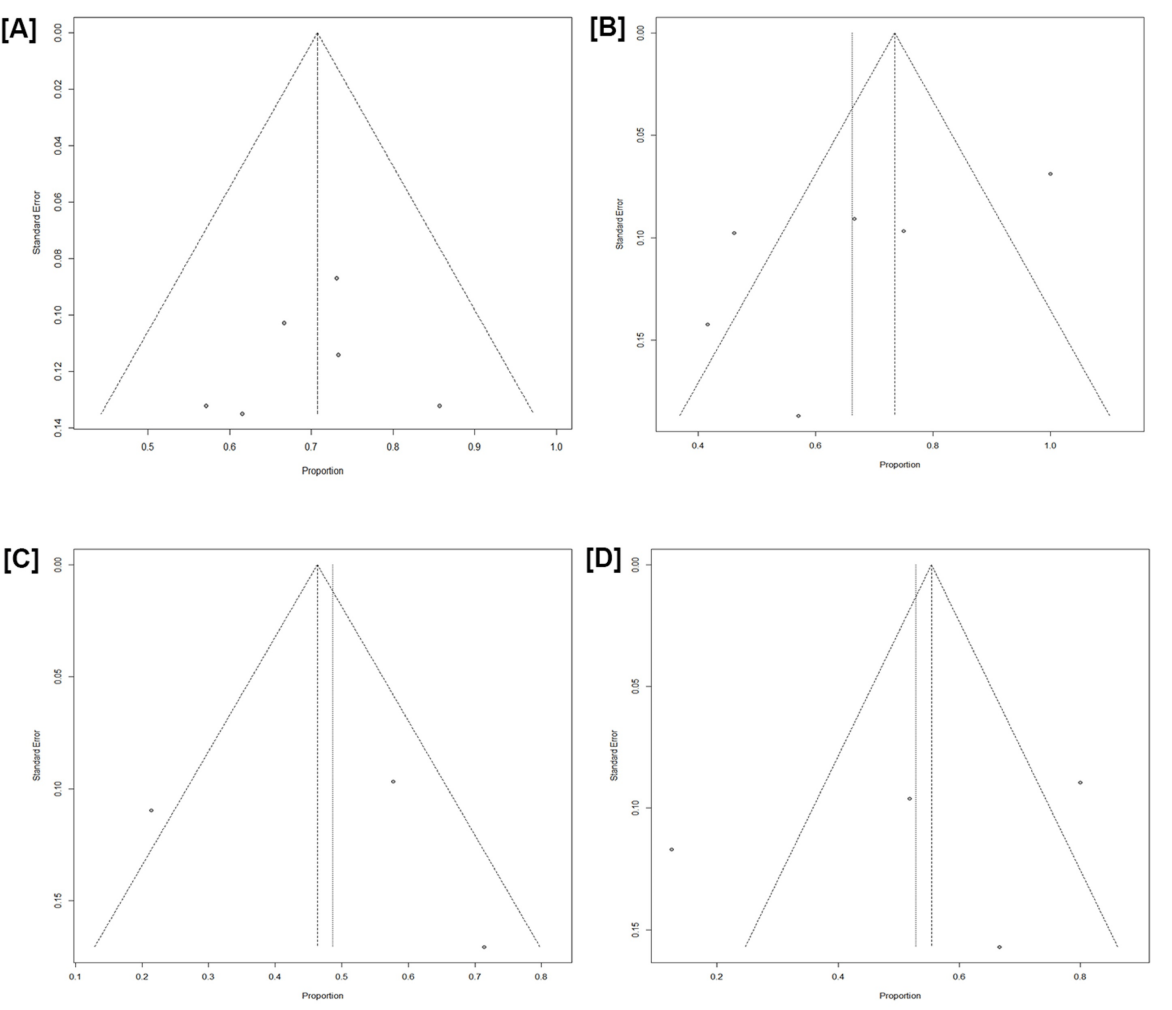
Funnel plot showing overall response (A), complete response (B), progression-free survival (C), and negative measurable residual disease (D)

**Figure 5:**
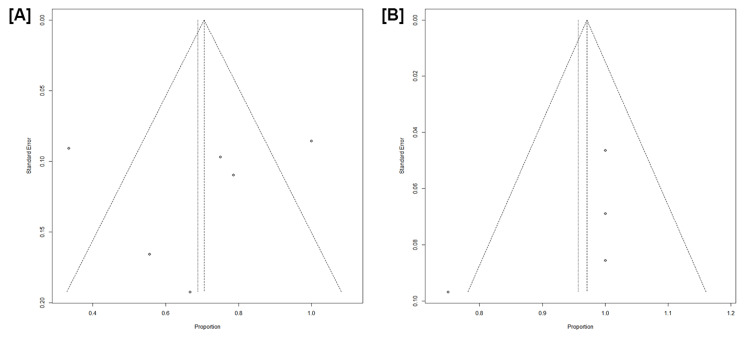
Funnel plot showing cytokine release syndrome (A) and immune effector cell-associated neurotoxicity syndrome (B)

The threat of a reporting bias was assessed through a visual inspection of the Deeks funnel plot and the Egger test. A funnel plot is a graphical tool commonly used in meta-analysis to assess publication bias and study heterogeneity. It plots individual study effect sizes against their precision or sample size, with smaller studies expected to be more spread out at the bottom and larger studies clustering at the top. A symmetrical funnel plot suggests no publication bias, while asymmetry may indicate that smaller studies with negative or null results are underrepresented. Additionally, the plot helps in the identification of heterogeneity by showing how consistently or variably studies report results. To create a funnel plot, the key metrics required include the effect size (or outcome measure) from each study, such as the odds ratio or mean difference, which is plotted on the x-axis. The study precision or sample size, often represented by the standard error (SE) or its inverse (1/SE), is plotted on the y-axis, with larger studies (higher precision) positioned near the top of the plot. The funnel plot for safety and efficacy is detailed in Figures 4, 5. Notably, except for overall response, the funnel plots displayed noticeable asymmetry, which suggests the presence of reporting bias. The Egger test analysis evaluates whether there is a relationship between the effect size and its SE, helping to detect potential publication bias. In the cases reviewed, the intercept values represent estimates of asymmetry in the funnel plot. For the Egger test analysis, the intercept values were as follows: for ORR, the intercept is 1.32 (CI: -3.96 to 6.60, p = 0.48), indicating no significant evidence of publication bias with a p-value greater than 0.05. Similarly, for CR, the intercept is 2.12 (CI: -7.48 to 11.74, p = 0.57), suggesting no evidence of publication bias. For PFS, the intercept is 0.02 (CI: -79.06 to 79.12, p = 0.99), also indicating no significant publication bias. For NMRD, the intercept value is -3.09 (CI: -23.77 to 17.58, p = 0.58), and likewise, the results suggest no publication bias. For CRS, the intercept is 4.30 (CI: -3.86 to 12.47, p = 0.21), which similarly suggests no evidence of publication bias. Finally, for ICANS, the intercept is 5.49 (CI: -10.17 to 21.15, p = 1.00), further confirming the absence of publication bias. In all cases, p-values greater than 0.05 suggest that there is no statistically significant evidence of publication bias.

Discussion

The meta-analysis presented here provides a comprehensive evaluation of the efficacy and safety of autologous/allogeneic DNA transposon-CD19 CAR T-cell therapy across 110 patients with relapsed/refractory B-ALL, B-NHL, and CLL. The findings underscore both the potential of this innovative approach and the challenges that need to be addressed for its widespread clinical adoption.

The pooled analysis revealed an ORR of 75%, indicating a substantial proportion of patients experiencing tumor regression following DNA transposon-CD19 CAR T-cell therapy. Furthermore, a significant percentage of patients (66%) achieved CR, demonstrating the potent anti-tumor activity of this treatment modality. These findings align with the meta-analysis demonstrating the efficacy of FDA-approved virus-generated CAR T-cell therapies, where ORR of axi-cel (axicabtagene ciloleucel), tisa-cel (tisagenlecleucel), and liso-cel (lisocabtagene maraleucel) in a heterogeneous group of patients with relapsed/refractory B-cell malignancies was reported as 77%, 69%, and 73% respectively [19]. The CR for axi-cel, tisa-cel, and liso-cel was reported as 52%, 57%, and 53%, respectively [19]. KTE-X19 (brexucabtagene autoleucel), another FDA-approved CD19 CAR T-cell therapy, reports a CR/CRi rate of 71% in R/R adult B-ALL patients [20]. Thus, the efficacy of DNA transposon-CAR T-cells is comparable to existing CAR T-cell therapies; however, the outcomes need to be validated in multicentric clinical trials with a larger cohort of patients. Sustained therapeutic response to the CAR T-cell therapy is often determined by the long-term persistence of CAR T-cells, which in turn is dependent upon the enrichment of the memory T-cell population in the infusion product and the differentiation of CAR T-cells toward memory T-cell phenotypes during in vivo expansion [21]. Despite longer manufacturing time, infusion products of DNA transposon-CD19 CAR T-cells are enriched with memory T-cells and thus capable of persisting long-term thereby inducing sustained remission [14,22].

Severe adverse events such as CRS or ICANS tend to occur rapidly within the first few weeks of treatment and can cause potentially life-threatening complications [1]. The DNA transposon-CD19 CAR T-cells were well tolerated at all dose levels tested in different clinical trials, and strikingly, no severe adverse events were directly attributed to this therapeutic platform. It is noteworthy that the rates of grade ≥3 CRS were absent among studies and 69% of patients treated with DNA transposon-CAR T-cells did not show any sign of grade 1/2 CRS. Similarly, the incidence of ICANS was not observed in 96% of patients treated with DNA transposon-CAR T-cells. Only few patients experienced grade 1/2 CRS and ICANS, which was manageable with small dosages of tocilizumab and corticosteroids, indicating that CD19 CAR T-cells generated using the DNA transposon systems exhibit a better safety profile in comparison to existing virus-generated CD19 CAR T-cell therapies. Axi-cel, tisa-cel, liso-cel, and KTE-X19 exhibit CRS of grade ≥3 in 9%, 21%, 2%, and 19% and ICANS in 31%, 8%, 10%, and 18% patients, respectively [19]. CRS developed as a result of the hyperactivation of immune effector cells following CAR T-cell infusion, which caused a massive surge of cytokines. However, ICANS, another common toxicity associated with CAR T-cell therapy, is a toxic encephalopathy state that presents with a wide range of neuropsychiatric symptoms [23,24]. Moderate levels of cytokine release after DNA transposon-CD19 CAR T-cell infusion could be one of the reasons for mild CRS and ICANS; however, the establishment of a plausible mechanism behind low levels of DNA transposon-CAR T-cell toxicity needs further investigation.

The incidence of secondary malignancies is being reported in long-term follow-ups of the patients treated with CAR T-cells, which varied between 4% and 16% [25]. It is suggested to occur because of CAR integration in regulatory regions of genes such as TET2, NFKB2, PTPRB, and/or JAK3 [25]. Emerging evidence suggests that the site of CAR integration is a key determinant of clonal expansion and T-cell malignancies [26]. Though transposon/transposase mediates a higher number of genomic integrations in comparison to the viral system, the integration of SB-CD19 CAR demonstrates no preference toward gene-dense regions, with no bias to target near promoters [16,22]. Moreover, CAR gene integration was maintained after the infusion of SB-CD19 CAR T-cells, suggesting that this therapeutic platform is safer than virus-generated CAR T-cell therapy; however, long-term follow-up of the patients is required to strengthen this finding [16]. Two cases of T-cell lymphoma were reported among the patients treated with PB-CD19 CAR T-cells [15]. An in-depth analysis of these patients demonstrates that, unlike the viral delivery system, the site of CAR gene integration into typical oncogene is apparently not a mechanism for PB-associated CAR T-cell malignancies. Instead, global changes in gene expression caused by transgene-mediated transcriptional readthrough along with genetic alterations such as point mutations, background genomic copy number variations, and high transgene copy number per cell have been proposed as possible mechanisms [27]. In addition, the likely mechanism by which the PB transposase induces oncogenesis has been linked to its prolonged activity. Furthermore, the PB transposase protein was detectable for more than one week, whereas the SB transposase was rapidly inactivated following gene delivery; thus, protein engineering may be required to solve the issue associated with PB transposase. These studies indicate that the SB transposon system may be a safer CAR gene delivery approach in comparison to PB and viral gene delivery systems.

Despite the promising efficacy and safety profiles demonstrated in this meta-analysis, the study is constrained by several limitations, which introduce significant heterogeneity into the data, thus limiting the robustness of the results. The small sample size precludes the possibility of performing a quantitative comparison between virus-generated CAR T-cell and DNA transposon-generated CAR T-cell therapies owing to the fact that an extensive body of evidence is available supporting the efficacy and safety of virus-generated CAR T-cells, whereas DNA transposon-based CAR T-cells are evaluated in early phases (Phase I/II) of clinical trials. Furthermore, the factors affecting the efficacy of CAR T-cell therapy such as the design of the CAR construct, patient characteristics, gene delivery system, and intricacies of the manufacturing process significantly varied among the studies included in the current meta-analysis, limiting our ability to draw definite conclusions. The availability of insufficient patient-level data further restricts us from performing subgroup analyses that would have allowed us to explore the impact of these variables on treatment outcomes. Another concern is the potential for publication bias, which is an inherent issue in the literature, particularly given the tendency of journals to favor the publication of studies reporting favorable outcomes, while negative or inconclusive findings are less frequently disseminated. Publication bias can lead to a skewed understanding of the true safety and efficacy of CAR T-cell therapies and may result in an overestimation of their therapeutic potential. The heterogeneity in the patient population, treatment protocols (e.g., autologous versus allogeneic), and outcome measures further complicate efforts to conduct a meaningful quantitative synthesis. Given these challenges, we adopted a conservative approach by pooling the data at the level of single rates. This approach allows us to synthesize the available data in a manner that acknowledges the inherent limitations of the studies included in this meta-analysis.

## Conclusions

The present meta-analysis provides evidence for a promising clinical response to DNA transposon-CD19 CAR T-cell therapy among patients with B-cell malignancies. Importantly, DNA transposon-CD19 CAR T-cells exhibited an anti-tumor response comparable to the virus-generated CD19 CAR T-cell therapy without the occurrence of severe adverse events. Thus, our study provides valuable insights into the efficacy and safety of DNA transposon-generated CAR T-cell therapy, highlighting its potential as a promising treatment modality for B-cell malignancies considering a cost-effective manufacturing process. While the current data demonstrates encouraging outcomes, additional well-designed, large-scale multicentric clinical trials are necessary to validate the long-term efficacy and safety of CAR T-cells generated using a non-viral approach. Future trials will help address any existing uncertainties, refine treatment protocols, and provide a more robust understanding of the therapeutic potential. Continued research is vital to ensure that the outcomes are reproducible and can be translated into effective, widely accessible therapy.

## Figures and Tables

**Figure 6:**
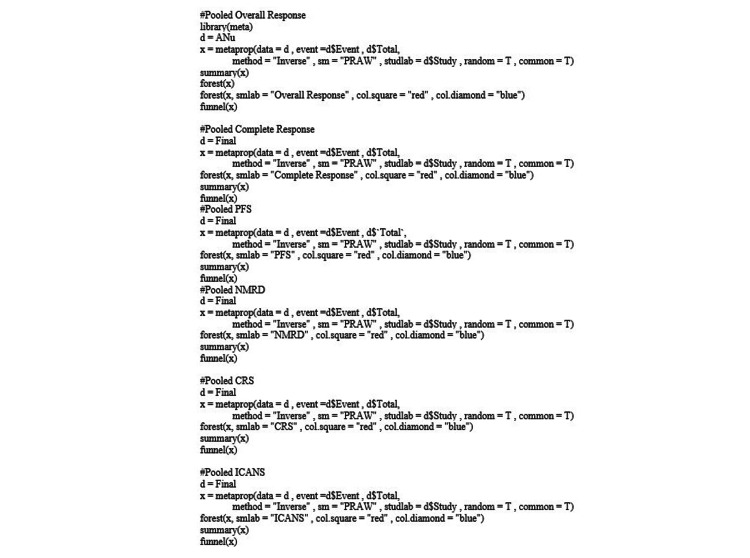
R software codes used to conduct the meta-analysis

## Appendices

Supplemental material 1

Supplemental material 2

**Table 3:** QUADAS-2: risk of bias assessment J: low risk of bias; L: high risk of bias; ?: unclear risk of bias

Study no.	Risk of bias				Applicability concerns		
	Patient selection	Index test	Reference standard	Flow and timing	Patient selection	Index test	Reference standard
Study 1 [12]	J	J	J	J	J	J	J
Study 2 [13]	J	J	J	J	J	J	J
Study 3 [14]	J	J	J	J	J	J	J
Study 4 [15]	J	L	J	J	J	J	J
Study 5 [16]	J	J	?	J	L	J	?
Study 6 [17]	J	J	L	J	J	J	J
Study 7 [18]	J	J	J	J	J	J	J

Supplemental material 3

**Table 4:** Cochran's Q test analysis Cochran’s Q test was used to assess statistically significant heterogeneity not explained by chance, whereas I2 was used to quantify the total observed variability due to between-study heterogeneity. I2 values > 50% were considered to indicate substantial heterogeneity. Statistical significance was established with an α level of 0.05 for primary analyses. CRS: cytokine release syndrome; ICANS: immune effector cell-associated neurotoxicity syndrome; NMRD: negative measurable residual disease

Overall response	
Test for heterogeneity	
Q	23.03
DF	4
Significance level	p = 0.0028
I^2^	72.7%
95% CI for I^2^	60% to 89.2%
Complete response	
Test for heterogeneity	
Q	28.97
DF	5
Significance level	p = <0.0001
I^2^	82.7%
95% CI for I^2^	63.6% to 91.8%
Progression-free survival	
Test for heterogeneity	
Q	8.69
DF	2
Significance level	p = 0.0130
I^2^	77.0%
95% CI for I^2^	25.0% to 92.9%
NMRD	
Test for heterogeneity	
Q	21.68
DF	3
Significance level	p = <0.0001
I^2^	86.20%
95% CI for I^2^	66.2% to 94.3%
CRS	
Test for heterogeneity	
Q	30.28
DF	5
Significance level	p < 0.0001
I^2^	83.50%
95% CI for I^2^	65.4% to 92.1%
ICANS	
Test for heterogeneity	
Q	5.89
DF	3
Significance level	p = 0.1169
I^2^	49.1%
95% CI for I^2^	0.00% to 83.1%
